# In Vivo Evaluation of Psoralen in a Copper Sulfate–Induced Chick (*Gallus gallus domesticus*) Emesis Model and In Silico Analysis of Its Interaction With D_2_, 5‐HT_3__A_, and Muscarinic Receptors

**DOI:** 10.1002/open.70267

**Published:** 2026-07-26

**Authors:** Kishor Chandra, Sharif Uddin Bahar, Khadija Akter, Mst Muslima Khatun, Samiran Sadhukhan, Progna Saha Puja, Fazley Rohan, Md Shimul Bhuia, Mohammed Alfaifi, Faisal H. Altemani, Faisal Alsenani, Salehin Sheikh, Muhammad Torequl Islam

**Affiliations:** ^1^ Department of Pharmacy Gopalganj Science and Technology University Gopalganj Bangladesh; ^2^ Bioinformatics and Drug Innovation Laboratory BioLuster Research Center Ltd. Gopalganj Bangladesh; ^3^ Department of Pharmaceutical Chemistry Netaji Subhas Chandra Bose Institute of Pharmacy Chakdaha West Bengal India; ^4^ Department of Clinical Laboratory Sciences College of Applied Medical Sciences King Khalid University Abha Saudi Arabia; ^5^ Department of Medical Laboratory Technology Faculty of Applied Medical Sciences University of Tabuk Tabuk Saudi Arabia; ^6^ Department of Pharmaceutical Sciences College of Pharmacy Umm Al‐Qura University Makkah Saudi Arabia; ^7^ Pharmacy Discipline Khulna University Khulna Bangladesh

**Keywords:** D_2_ and 5‐HT_3_ receptors, emesis, in vivo chick emesis model, molecular docking, psoralen

## Abstract

Psoralen (PSN), a naturally occurring furocoumarin, was evaluated for its antiemetic potential using integrated in vivo and in silico approaches, considering the limitations of currently available antiemetic agents. The antiemetic activity of PSN was investigated in a copper sulfate–induced emesis model using 2‐day‐old chicks (*Gallus gallus domesticus*). A dose‐dependent experimental design was employed with PSN (5, 10, and 20 mg/kg), alongside standard antiemetics (domperidone 6 mg/kg, ondansetron (OND) 5 mg/kg, and hyoscine 21 mg/kg) and combination treatments. Latency to first retch and total retching episodes were recorded, and percentage inhibition was calculated. Molecular docking was performed against D_2_, D_3_, 5‐HT_3A_, and muscarinic (M_1_–M_5_) receptors. ADMET and toxicity profiles were predicted using SwissADME and ProTox‐3.0. PSN produced a significant, dose‐dependent reduction in retching. The 20 mg/kg dose showed 65.60% inhibition of retches compared to control. Combination treatment with OND further enhanced inhibition (65.60%). Docking analysis revealed that PSN exhibited the strongest binding affinity toward the D_2_ receptor (−8.8 kcal/mol), followed by M_5_ (−8.1 kcal/mol) and 5‐HT_3A_ (−7.7 kcal/mol). Pharmacokinetic prediction indicated high gastrointestinal absorption and favorable drug‐likeness properties. PSN demonstrates significant antiemetic activity in a validated chick emesis model, supported by moderate binding affinity toward key emesis‐related receptors. While docking findings suggest possible receptor interactions, further mechanistic and translational studies are required to confirm the molecular basis of its activity.

## Graphical abstract

Psoralen (PSN) demonstrates significant dose‐dependent antiemetic activity in a copper sulfate–induced chick emesis model. Integrated in vivo and in silico analyses reveal reduced retching frequency, prolonged latency, and favorable multi‐receptor interactions with D_2_, 5‐HT_3_A, and muscarinic receptors, highlighting PSN as a promising multi‐target antiemetic candidate with favorable drug‐likeness properties.

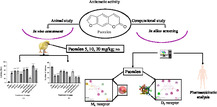

## 1 Introduction

Vomiting, or emesis, the violent ejection of stomach contents, is a typical physiological reaction that may be triggered by a range of central and peripheral stimuli [1]. The uncomfortable, painless, and subjective sensation of feeling like one is ready to throw up is known as “nausea.” In contrast, “vomiting” refers to the coordinated sequence of muscular and autonomic reflexes that contribute to the violent evacuation of what is in the stomach through the mouth [2]. The problem is that there are numerous reasons for nausea and vomiting, and treating certain people may be difficult [3]. Various disorders, such as food‐related poisoning, gastroenteritis (diarrhea), motion sickness, hangovers, cranial trauma, intestinal blockage, appendicitis, high intracranial pressure, and postoperative problems, can also cause vomiting and nausea in addition to absorbing poisons or irritants [4]. Over 90% of the body's serotonin (5‐HT) and a significant amount of substance P (SP), both of which play crucial roles in digestion, nausea, and vomiting, are produced by enterochromaffin cells located in the gut. These cells release 5‐HT and/or SP in response to various emetogenic stimuli, which can be chemical, mechanical, or neurological. This release process is dependent on calcium (Ca^2+^). Once released, 5‐HT and likely SP activate their corresponding emetic receptors on the vagal efferent, specifically the substance P neurokinin NK1 receptors and serotonin 5‐HT_3A_ receptors, leading to the sensations of nausea and vomiting [5]. Dopamine receptors also play an important role in inducing emesis [6].

The emetic reflex arc is orchestrated by a network of brainstem nuclei. Key among these are the nucleus tractus solitarius (NTS) and the chemoreceptor trigger zone (CTZ), located in the area postrema [7]. Due to its location outside the blood‐brain barrier, the CTZ can directly sense emetogenic agents in the bloodstream and cerebrospinal fluid. Peripheral stimuli, such as gastrointestinal irritants, activate vagal afferent nerves, which project to the NTS. A complex interplay of neurotransmitters, including serotonin (5‐HT) acting on 5‐HT_3_ receptors, dopamine on D_2_ receptors, and acetylcholine on muscarinic receptors, mediates these signals within the emetic circuitry [8].

Current pharmacotherapy for nausea and vomiting includes antagonists of these key receptors. For instance, ondansetron (OND) is a selective 5‐HT_3A_ receptor antagonist, domperidone (DOM) blocks D_2_ receptors, and hyoscine (scopolamine) is a muscarinic receptor antagonist. While effective, these agents are not without limitations. Their use can be associated with adverse effects such as extrapyramidal symptoms (with metoclopramide, a D_2_ antagonist), QT interval prolongation (with OND), and anticholinergic effects (with hyoscine) [9]. Furthermore, given the multi‐pathway nature of emesis, single‐target therapies may provide incomplete symptom control, often necessitating the use of combination drug regimens. This highlights the ongoing need for novel antiemetic agents with favorable safety profiles and the potential for multi‐receptor modulation.

Psoralen (PSN), chemically designated as furo[3,2‐g]chromen‐7‐one, is a naturally occurring furanocoumarin widely recognized for its diverse pharmacological properties. Traditionally known as “Buguzhi” in Chinese medicine, PSN is the principal bioactive constituent of the dried fruits of *Cullen corylifolium* (L.) Medik (*Psoralea corylifolia* L.). First reported in 1971, PSN is well known for its photoreactive properties, forming DNA interstrand crosslinks upon ultraviolet A (UVA) exposure, a mechanism that underlies its application in PUVA therapy as well as its mutagenic potential [10]. Beyond its photochemical properties, PSN has demonstrated a broad spectrum of biological activities, including anti‐inflammatory, antiviral, antibacterial, antitumor, anti‐osteoporotic, antioxidant, and neuroactive effects [11]. Notably, it has been shown to regulate bone homeostasis by modulating osteoblast, osteoclast, and chondrocyte differentiation and activity, supporting its therapeutic relevance in osteoporosis.

Emerging evidence suggests that PSN and structurally related coumarins can influence neurotransmitter signaling pathways, particularly serotonergic and dopaminergic systems. The planar aromatic furanocoumarin scaffold of PSN is conducive to receptor–level interactions, enabling favorable Pi–Pi stacking and hydrophobic contacts within ligand‐binding domains, including those of G protein‐coupled receptors (GPCRs). These structural and pharmacodynamic features provide a mechanistic rationale for investigating PSN as a neuromodulatory agent capable of interacting with receptor systems implicated in neurophysiological regulation.

Despite extensive pharmacological characterization of PSN, its potential role in emesis modulation has not been systematically explored. No prior experimental or computational studies have evaluated PSN as an antiemetic agent or examined its interaction with key emetic receptors such as D_2_, 5‐HT_3A_, or muscarinic subtypes. Given its documented anti‐inflammatory and neuromodulatory properties, together with structural features favorable for multi‐receptor engagement, PSN represents a plausible candidate for emetic regulation.

Therefore, the present study aimed to evaluate the antiemetic potential of PSN using a copper sulfate‐induced chick emesis model and to investigate its interactions with major emesis‐related receptors (D_2_, 5‐HT_3_, and muscarinic subtypes) through in silico molecular docking. This work provides the first integrated in vivo and in silico assessment of PSN as a candidate antiemetic compound, extending its pharmacological profile and introducing a novel translational perspective as a potential multi‐target modulator of emetic pathways.

## 2 Materials and Methods

### 2.1 Chemicals and Reagents

PSN (CAS: 66‐97‐7, 99% HPLC purity) was bought from Centurion Healthcare Private Ltd., Chengdu, China 610000, while copper sulfate pentahydrate (CuSO_4_⋅5H_2_O) was obtained from Merck (India). Standard drugs, OND, DOM, and hyoscine butyl bromide (HYS) were purchased from Incepta, Beximco, Opsonin, Beacon, and Eskayef Pharma Ltd., Bangladesh, respectively.

### 2.2 Selection and Preparation of Test and Control Groups

We selected the test sample at three concentrations: 5, 10, and 20 (lower, middle, and higher) after reviewing the literature [12]. The mother solution was made by dissolving the sample in distilled water (DW) and applying an adequate amount of Tween 80 (0.5%) as a solvent. The mother solution's concentration was 50 mg/kg. It was then diluted at 20, 10, and 5 mg/kg concentrations. On the other hand, the dosages of the medications that were referred to were selected by converting human dosages to animal dosages that were backed by literature processes and animal dose calculation protocols [13]. The reference drug's solutions were also prepared by fully combining them with DW at concentrations of 6, 21, and 5 mg/kg for the medications DOM, HYS, and OND, respectively. A tiny quantity of Tween 80 was utilized as a cosolvent in this process. For the co‐treatments, three combined dosages of DOM, HYS, OND, and PSN (20 mg/kg) were also made.

### 2.3 Experimental Animals

Two‐day‐old domestic chicks (*Gallus gallus domesticus*), mixed sex, weighing 40–45 g, were obtained from a commercial hatchery (Rupsha Poultry and Hatchery Ltd., Bangladesh). The chicks were not of a defined genetic strain, as they were procured from a local commercial source for experimental pharmacological screening. For this investigation, all of the chicks were housed at Gopalganj Science and Technology University's pharmacology lab in Gopalganj. The chicks had unrestricted access to normal food and water. A 12‐h dark/light cycle and controlled illumination were used to keep them at 27 ± 2 °C before the test. After fasting for 12 h, the antiemetic test was performed. The Ethical Committee and the Pharmacy Department of Gopalganj Science and Technology University gave their approval to this study (#GSTU‐pharmacy‐19PHR022). Furthermore, every method was used in compliance with applicable rules and regulations, and every method was reported in compliance with ARRIVE standards.

### 2.4 In Vivo Protocol

Since chicks are capable of vomiting, a direct emesis model was employed. A pica (kaolin consumption) model was not applicable, as it is primarily used in rodent species that lack the vomiting reflex. The experimental protocol was slightly adapted from the method outlined by Akita et al. [14]. The study involved a total of nine groups, each comprising five chicks. Chicks were randomly allocated to treatment groups using a simple random assignment method. The investigator responsible for recording latency and retching frequency was blinded to the treatment groups to minimize observational bias. Before administering any treatment, each chick was placed individually in a large, transparent plastic chamber for 10 min to acclimatize. The test compound, PSN, was dissolved in DW and given orally at doses of 5, 10, and 20 mg/kg. For comparison, standard drugs DOM, HYS, and OND were administered orally at doses of 6, 21, and 5 mg/kg, respectively. Additionally, to examine potential synergistic or antagonistic interactions, combinations of PSN (20 mg/kg) with each reference drug (DOM, HYS, and OND) were also prepared and administered orally. The control group received only the vehicle solution, consisting of DW with 0.5% Tween 80. All oral treatments were delivered in a uniform volume of 10 mL/kg body weight. After 30 min of treatment, emesis was induced by orally administering copper sulfate pentahydrate (CuSO_4_ · 5H_2_O) at a dose of 50 mg/kg. For each chick, researchers recorded the latency to the first retch (i.e., the time interval between CuSO_4_ · 5H_2_O administration and the first observed retching) and the total number of retches occurring within a 10 min observation period. They calculated the percentage inhibition of retching and the increase in latency relative to the control group using standard formulas.

Details of the treatment groups and their corresponding doses are presented in Table 1.

**TABLE 1:** Name of treatment groups, their dose, and target receptor.

Treatment group	Description	Activity pathway
Gr‐1: NC	0.5% Tween 80 dissolved in DW	—
Gr‐2: PSN‐5	Psoralen, 5 mg/kg	Under investigation
Gr‐3: PSN‐10	Psoralen, 10 mg/kg	Under investigation
Gr‐4: PSN‐20	Psoralen, 20 mg/kg	Under investigation
Gr‐5: OND‐5	Ondansetron	5‐HT_3A_ receptor antagonist
Gr‐6: DOM‐6	Domperidone	D_2_ dopamine receptor antagonist
Gr‐7: HYS‐21	Hyoscine butyl bromide	Muscarinic (M_1_–M_5_) receptor antagonist
Gr‐8: PSN‐20 + OND‐5	Psoralen‐20 mg/kg and ondansetron	Under investigation
Gr‐9: PSN‐20 + DOM‐6	Psoralen‐20 mg/kg and domperidone	Under investigation
Gr‐10: PSN + HYS21	Psoralen 20 mg/kg and hyoscine	Under investigation

$$\%\;\text{Decrease}\;\mathrm{in}\;\text{retches}=\frac{C-D}{C}\times 100$$

where *C* represents the vehicle group's mean retches, and *D* represents the standard and test group's mean retches.

### 2.5 Acute Toxicity and Safety Observation

To assess preliminary safety, chicks treated with the highest dose of PSN (20 mg/kg) were observed for 24 h for signs of behavioral abnormalities, mortality, or overt toxicity. No mortality or visible signs of acute toxicity were observed during the observation period.

### 2.6 Statistical Analysis

Data were expressed as mean ± SEM (*n* = 5 per group). Statistical analysis was performed using GraphPad Prism (version 10.4.2.633, GraphPad Software Inc., USA). Data distribution was assessed for normality using the Shapiro–Wilk test, and homogeneity of variances was evaluated using Levene's test. As the assumptions of normality and equal variance were satisfied, group comparisons were conducted using one‐way analysis of variance (ANOVA), followed by Dunnett's post hoc test for multiple comparisons, specifically comparing each treatment group with the normal control (NC) group. This approach was selected to control the family‐wise error rate while restricting comparisons to biologically relevant treatment‐versus‐control contrasts. A value of *p* < 0.05 was considered statistically significant.

*Power analysis:* A post hoc power analysis was conducted using one‐way ANOVA (statsmodels package in Python). Based on the observed between‐group variability, the calculated effect size (Cohen's *f* = 1.73) indicated a very large treatment effect. The estimated statistical power exceeded 0.99 at *α* = 0.05, demonstrating that a sample size of five animals per group was sufficient to detect biologically and statistically significant differences among treatment groups.

### 2.7 In Silico Analysis

#### 2.7.1 Selection and Preparation of Receptors

To conduct molecular docking and ligand‐receptor visualization, eight emesis‐causing receptors were chosen for this investigation based on the literature review. On June 1, 2025, the RCSB Protein Data Bank (https://www.rcsb.org/) provided the three‐dimensional (3D) structures of 5‐HT_3A_ (PDB ID: 6NP0), D_2_ (PDB ID: 6CM4), D_3_ (PDB ID: 3PBL), M_1_ (PDB ID: 6WJC), M_2_ (PDB ID: 5ZK8), M_3_ (PDB ID: 4U15), M_4_ (PDB ID: 5DSG), and M_5_ (PDB ID: 6OL9). After that, the structures went through optimization procedures to lessen any docking interference. PyMOL (v3.1.4.1) was used to remove lipids, water molecules, and heteroatoms from the structures. Invoking the GROMOS96 force field, the SwissPDB Viewer was finally utilized. It reduced energy and optimized the receptor, which was saved as a PDB file to execute molecular docking.

#### 2.7.2 Collection and Preparation of Ligands

**FIGURE 1:**
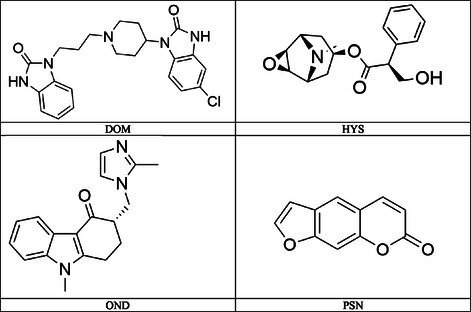
Chemical structures of the investigated compounds. The figure illustrates the molecular structures of psoralen (PSN), the test compound evaluated for antiemetic activity, and the reference antiemetic drugs: domperidone (DOM, dopamine D_2_ receptor antagonist), ondansetron (OND, 5‐HT_3_ receptor antagonist), and hyoscine (HYS, muscarinic receptor antagonist). Structural comparison highlights differences in molecular size, aromaticity, and functional groups that may influence receptor–binding interactions and pharmacological activity.

3D conformers of Granisetron (GRN), Risperidone (RPD), Eticlopride (ETI), Benzeneacetic acid (BAA), N‐methyl scopolamine (NMS), Tiotropium (TIO), DOM (Compound CID: 3151), HYS (Compound CID: 3000322), OND (Compound CID: 4595), and PSN (Compound CID: 6199), were collected in SDF format from the PubChem chemical database (https://pubchem.ncbi.nlm.nih.gov/, accessed on 01 June 2025). Then, to perform molecular docking and PK prediction, we used the Chem3D 21.0 computer application, which reduced the chemical compounds’ 3D conformers and stored them in SDF format. Prior to molecular docking, the PyRx program was used to optimize both ligands and molecules. Figure 1 depicts the chemical structures of both PSN and standard drugs.

#### 2.7.3 Molecular Docking and Prediction of Ligand–Receptor Interactions

The PyRx software application was used to perform molecular docking in order to predict the active binding sites of medicines against receptors. The computation was carried out in 200 steps, with an exhaustiveness value of 20. Active binding sites and corresponding grid box parameters were determined using the PrankWeb server based on predicted pocket residues and binding scores [15]. The ligand–protein complex is stored in PDB format for ligand collection in PDBQT, and the docking potential is exported in “CSV” format. The Discovery Studio Visualizer (v21.1.020298) and PyMoL (v3.1.4.1) program packages were used to observe the interactions between ligand‐receptors and the receptor's active site. The list includes the amino acid residues, bond types, number of hydrogen bonds, and length of hydrogen bonds of each ligand–receptor interaction.

#### 2.7.4 Docking Validation

Docking validation was performed to evaluate the reliability and reproducibility of the molecular docking protocol. The native co‐crystallized ligands of each receptor were extracted from their respective protein structures and subsequently redocked into the original binding pocket using the same docking parameters employed for the test compounds. Validation accuracy was assessed by calculating the root mean square deviation (RMSD) between the experimentally observed co‐crystal pose and the redocked pose after structural superimposition. An RMSD value below 2.0 Å was considered indicative of a reliable docking protocol and acceptable reproduction of the experimental binding orientation [16].

#### 2.7.5 Prediction of Drug‐Likeness and Pharmacokinetics

Drug likeness is a quantitative measurement that is used in drug development in advance to eliminate drugs that can fail in clinical trials and to reduce cost. The pharmacokinetics and drug‐likeness of a chemical agent can be calculated utilizing a number of web servers and programs. The current study used a web‐based ADME prediction tool called SwissADME to forecast the pharmacokinetic and drug‐like properties of the test and standard compounds (http://www.swissadme.ch/index.php, accessed on June 1, 2025) [17].

#### 2.7.6 Toxicity Prediction

To predict toxicity parameters, we used the ProTox‐3.0 web servers. ProTox‐3.0 employs powerful machine learning models to provide insights into target organ toxicity, toxicological endpoints, and LD50 estimates [18]. We input the canonical SMILES of compounds from PubChem into the ProTox‐3.0 search bar to predict their toxicity.

## 3 Results

### 3.1 In Vivo Result

Copper sulfate administration produced a marked emetic response in the negative control (NC) group, characterized by the shortest latency period (5 ± 1.30 s) and the highest frequency of retching (31.4 ± 2.33).

Treatment with standard antiemetic agents significantly attenuated emesis. DOM (6 mg/kg) prolonged latency to 45.8 ± 2.59 s (*p* < 0.0001) and reduced retching frequency to 13.6 ± 1.71 (*p* < 0.0001), corresponding to 56.68% inhibition relative to the NC group. OND (5 mg/kg) increased latency to 57.2 ± 3.76 s (*p* < 0.0001) and reduced retching to 14.8 ± 1.98 (*p* < 0.0001) (52.86% inhibition). HYS (21 mg/kg) demonstrated comparatively lower inhibition, reducing retches to 23 ± 1.92 (*p* = 0.0248) (26.75% inhibition), with a latency of 55.8 ± 5.14 s (*p* < 0.0001).

PSN exhibited dose‐dependent antiemetic activity. PSN‐5 reduced retching to 23.2 ± 2.68 (*p* = 0.0299) (26.11% inhibition) and prolonged latency to 42.6 ± 2.33 s (*p* < 0.0001). PSN‐10 further decreased retching to 20.4 ± 1.32 (*p* = 0.0018) (35.03% inhibition), with a latency of 62 ± 2.44 s (*p* < 0.0001). Notably, PSN‐20 produced the most pronounced effect, reducing retching to 10.8 ± 1.23 (*p* < 0.0001), corresponding to 65.60% inhibition relative to NC, and extending latency to 64.4 ± 2.65 s (*p* < 0.0001). The inhibitory effect of PSN‐20 exceeded that observed with DOM (56.68%) and OND (52.86%) in this experimental model.

**FIGURE 2:**
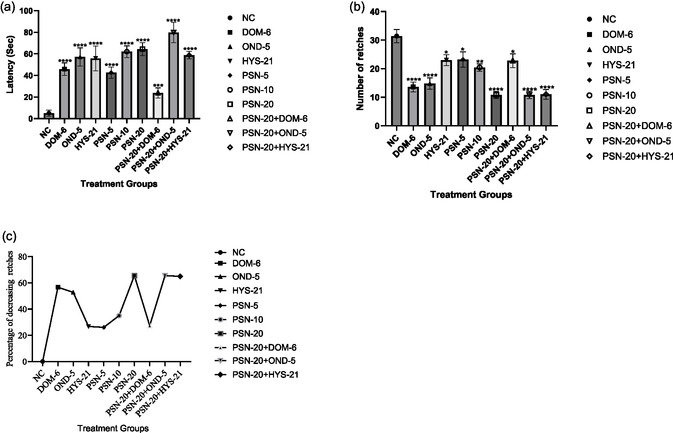
(a) Latency (sec), (b) number of retches, and (c) percentage of decreasing retches. Comparison of experimental groups analyzed using one‐way ANOVA followed by Dunnett's post hoc test, where all treatment groups were compared only against the control group. Data are presented as mean ± SEM (*n* = 5). Statistical significance is indicated as follows: *p* < 0.05 (*), *p* < 0.01 (**), *p* < 0.001 (***), and *p* < 0.0001 (****) [PSN: psoralen, DOM: domperidone, OND: ondansetron].

Combination therapy further influenced the antiemetic response. PSN‐20 + OND‐5 reduced retching to 10.8 ± 1.27 (*p* < 0.0001) (65.60% inhibition) and produced the longest latency period (79.8 ± 4.21 s) (*p* < 0.0001). PSN‐20 + HYS‐21 resulted in 11 ± 1.69 retches (*p* < 0.0001) (64.96% inhibition) with a latency of 58.8 ± 1.55 s (*p* < 0.0001). In contrast, PSN‐20 + DOM‐6 demonstrated comparatively lower inhibition 27.38%; retches (22.8 ± 2.43) (*p* = 0.0206) and a shorter latency (23.8 ± 2.12 s) (*p* = 0.0007). Figure 2 shows the latency, b) number of retches, and percentage of decreased retches.

Overall, PSN demonstrated dose‐dependent antiemetic activity, with PSN‐20 showing the greatest reduction in retching frequency and a prolonged latency period compared to both the NC and standard antiemetic drugs.

### 3.2 In Silico Study

#### 3.2.1 Molecular Docking Study

The molecular docking analysis provided comparative insights into the binding affinities of the test compound PSN with co‐crystallized ligands and standard antiemetic drugs across multiple emesis‐related receptors (Table 2). The active binding pockets and grid center coordinates used for docking were predicted using the PrankWeb server and are summarized in Table 3. For the 5‐HT_3_ receptor, PSN exhibited a binding affinity of −7.7 kcal/mol, which was identical to the standard drug OND (−7.7 kcal/mol) but slightly lower than the co‐crystal ligand GRN (−8.3 kcal/mol). This suggests that PSN may possess comparable interaction potential to clinically used 5‐HT_3_ antagonists.

**TABLE 2:** Molecular docking scores (kcal/mol) of psoralen and selected reference standards.

Receptors Ligands	5‐HT_3A_	D_2_	D_3_	M_1_	M_2_	M_3_	M_4_	M_5_
Granisetron (co‐crystal)	−8.3	—	—	—	—	—	—	—
Ondansetron	−7.7	—	—	—	—	—	—	—
Risperidone (co‐crystal)	—	−12	—	—	—	—	—	—
Eticlopride (co‐crystal)	—	—	−6.7	—	—	—	—	—
Domperidone	—	−11.3	−9.8					
Benzeneacetic acid (co‐crystal)	—	—	—	−6.9	—	—	—	—
N‐methyl scopolamine (co‐crystal)	—	—	—	—	−7.6	—	—	—
Tiotropium (co‐crystal)	—	—	—	—	—	−8.3	−8.0	−8.3
Hyosine butyl bromide	—	—	—	−6.7	−7.2	−8.0	−7.9	−7.5
Psoralen	−7.7	−8.8	−6.9	−7.6	−7.9	−7.1	−8.3	−8.1

**TABLE 3:** Binding pocket prediction of target proteins using the PrankWeb.

PDB ID	Rank	Probability	Pocket center	Amino acid residues
6NP0	1	0.873	(147.4447, 152.3111, 168.6639)	A_256, A_259, A_260, A_263, A_264, A_266, A_267, A_269, A_270, A_273, A_274, A_275, A_280, A_281, A_283, A_284, A_287, A_288, A_291, E_225, E_226, E_229, E_230, E_233, E_234, E_258, E_261, E_265
6CM4	1	0.888	(9.3302, 6.1417, −10.1226)	A_100, A_110, A_114, A_115, A_118, A_119, A_184, A_189, A_190, A_193, A_197, A_198, A_386, A_389, A_390, A_393, A_408, A_409, A_412, A_413, A_416, A_91, A_94, A_95
3PBL	1	0.848	(−0.2976, −16.0790, 10.9225)	A_106, A_107, A_110, A_111, A_114, A_181, A_182, A_183, A_189, A_192, A_193, A_196, A_342, A_345, A_346, A_349, A_350, A_365, A_366, A_369, A_373, A_86, A_89, A_90, A_94
6WJC	1	0.884	(20.4523, 13.6905, 3.3277)	A_101, A_102, A_105, A_106, A_109, A_110, A_113, A_157, A_178, A_179, A_180, A_183, A_186, A_189, A_192, A_193, A_196, A_197, A_378, A_381, A_382, A_385, A_388, A_389, A_400, A_404, A_407, A_408, A_74, C_31, C_32, C_33, C_34, C_35
5ZK8	1	0.913	(184.0322, 27.7125, 522.3441)	A_100, A_103, A_104, A_107, A_108, A_110, A_155, A_176, A_177, A_178, A_181, A_187, A_190, A_191, A_194, A_195, A_400, A_403, A_404, A_407, A_410, A_419, A_422, A_423, A_426, A_429, A_430, A_432, A_433, A_69, A_72, A_80, A_83, A_84, A_99
4U15	1	0.953	(31.6568, 103.1821, 51.1334)	A_198, A_201, A_202, A_206, A_209, A_210, A_214, A_223, A_224, A_226, A_227, A_230, A_234, A_237, B_105, B_152, B_155, B_156, B_159, B_160, B_163, B_164, B_167, B_171, B_175, B_178, B_179, B_180, B_183, B_184, B_187, B_190, B_194, B_198, B_237, B_238, B_241, B_242, B_245
5DSG	1	0.946	(49.4383, 11.5366, 63.2987)	A_108, A_109, A_112, A_113, A_116, A_117, A_164, A_184, A_185, A_186, A_187, A_190, A_191, A_196, A_199, A_200, A_203, A_204, A_413, A_416, A_417, A_420, A_423, A_432, A_435, A_436, A_439, A_442, A_443, A_89, A_92, A_93
6OL9	1	0.899	(34.5368, 24.8097, −36.9640)	A_106, A_107, A_110, A_111, A_114, A_115, A_162, A_182, A_183, A_184, A_185, A_188, A_189, A_191, A_194, A_197, A_198, A_201, A_455, A_458, A_459, A_462, A_465, A_470, A_472, A_474, A_477, A_478, A_481, A_484, A_485, A_87, A_90

In the case of the dopaminergic receptors, PSN demonstrated a moderate binding affinity toward the D_2_ receptor (−8.8 kcal/mol), which, although lower than both the co‐crystal ligand RPD (−12 kcal/mol) and the standard DOM (−11.3 kcal/mol), still indicates a favorable interaction. Similarly, for the D_3_ receptor, PSN showed a binding affinity of −6.9 kcal/mol, slightly better than the co‐crystal ligand ETI (−6.7 kcal/mol) but weaker than DOM (−9.8 kcal/mol). These findings indicate that PSN can interact with dopaminergic receptors, albeit with lower affinity compared to standard antagonists.

Regarding the muscarinic receptor subtypes, PSN exhibited notable binding affinities across all receptors (M_1_–M_5_). For the M_1_ receptor, PSN (−7.6 kcal/mol) showed stronger binding than both the co‐crystal ligand BAA (−6.9 kcal/mol) and the standard HYS (−6.7 kcal/mol). Similarly, for the M_2_ receptor, PSN (−7.9 kcal/mol) demonstrated slightly higher affinity than both the co‐crystal ligand NMS (−7.6 kcal/mol) and HYS (−7.2 kcal/mol). For the M_3_ receptor, PSN (−7.1 kcal/mol) displayed weaker binding compared to both TIO (−8.3 kcal/mol) and HYS (−8.0 kcal/mol). In contrast, PSN showed superior binding affinity for the M_4_ receptor (−8.3 kcal/mol) compared to tiotropium (−8.0 kcal/mol) and hyoscine (−7.9 kcal/mol). For the M5 receptor, PSN (−8.1 kcal/mol) demonstrated comparable affinity to the co‐crystal ligand tiotropium (−8.3 kcal/mol) but higher than hyoscine (−7.5 kcal/mol).

Overall, these results indicate that PSN may exhibit multi‐receptor binding capability, with particularly strong interactions toward muscarinic receptors (especially M_4_ and M_5_) and moderate affinity toward dopaminergic and serotonergic receptors. Although its binding affinity is generally lower than high‐affinity co‐crystal ligands and standard drugs for certain receptors, PSN demonstrates a balanced and broad–spectrum interaction profile, which may contribute to its observed antiemetic activity.

The docking protocol was validated through redocking of the native co‐crystallized ligands into the active sites of all selected receptors. The reproduced binding conformations demonstrated RMSD values ranging from 0.151 to 1.661 Å, all below the commonly accepted threshold of 2.0 Å (Table 4). Structural superimposition analysis further confirmed close overlap between the experimental and redocked ligand poses, indicating satisfactory accuracy and reproducibility of the docking parameters employed in the present study.

**TABLE 4:** Docking validation by superimposition of native (red) and redocked (blue) ligand poses within receptor binding sites.

SL No.	PDB ID	Co‐crystal ligand	RMSD	Superimposition
1	6NP0	Granisetron	0.326	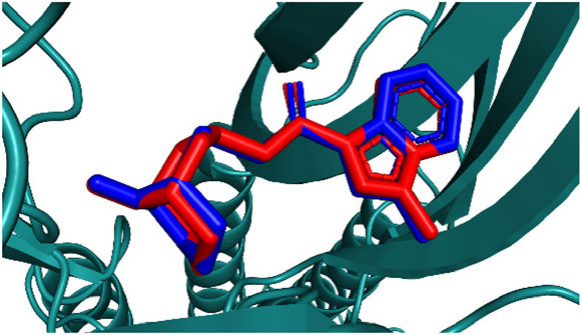
2	6CM4	Risperidone	1.661	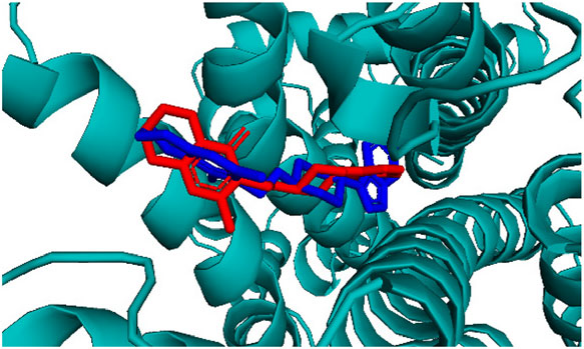
3	3PBL	Eticlopride	0.925	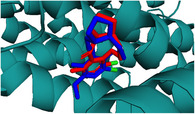
4	6WJC	Benzeneacetic acid	0.465	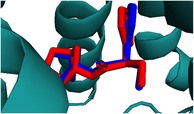
5	5ZK8	N‐methyl scopolamine	1.295	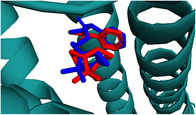
6	4U15	Tiotropium	0.788	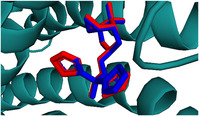
7	5DSG	Tiotropium	0.151	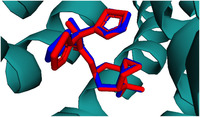
8	6OL9	Tiotropium	1.131	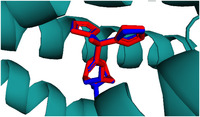

#### 3.2.2 Prediction of Nonbond Interactions Between Drug–Receptor Complexes

The nonbond interaction analysis further elucidated the binding modes of PSN in comparison with co‐crystal ligands and standard drugs, focusing on hydrogen bond (HB), bond lengths, and hydrophobic interactions (Table 5).

**TABLE 5:** Amino acid residues, number of hydrogen bonds and hydrogen bond length of nonbond interactions between the selected ligands and receptors.

Receptors	Ligands	No. of HB	HB residues	HB length, Å	Other bond residues
5‐HT_3A_	Granisetron	—	—	—	A:ARG65 (Pi‐Cation) A:ILE44 (Alkyl) A:ARG65 (Alkyl) A:ILE112 (Alkyl) A:PRO128 (Alkyl) A:ILE44 (Pi‐Alkyl) A:ARG65 (Pi‐Alkyl) A:TRP63 (Pi‐Alkyl) A:TYR126 (Pi‐Alkyl)
	Ondansetron	—	—	—	A:ARG65 (Pi‐Cation) A:TYR126 (Pi‐Sigma) A:ILE44 (Pi‐Sigma) A:TYR126 (Pi–Pi Stacked) A:ARG65 (Pi‐Alkyl) A:ILE44 (Pi‐Alkyl) A:TRP63 (Pi‐Alkyl)
	Psoralen	2	A:TYR64 A:LYS127	2.95711, 2.8885	A:ARG65 (Pi‐Cation) A:ILE44 (Pi‐Sigma) A:TRP63 (Pi‐Sigma) A:TYR126 (Pi–Pi T‐shaped) A:ILE44 (Pi‐Alkyl) A:ARG65 (Pi‐Alkyl)
D_2_	Risperidone	1	A:SER197	3.55922	A:CYS118 (Halogen‐Fluorine) A:THR412 (Pi‐Sigma) A:TRP100 (Pi–Pi T‐shaped) A:TRP386 (Pi–Pi T‐shaped) A:VAL91 (Alkyl) A:LEU94 (Alkyl) A:ILE184 (Alkyl) A:TRP100 (Pi‐Alkyl) A:PHE389 (Pi‐Alkyl) A:TRP413 (Pi‐Alkyl) A:CYS118 (Pi‐Alkyl) A:VAL115 (Pi‐Alkyl)
	Domperidone	5	A:THR119 A:ASP114 A:ASP114 A:ASP114 A:THR412	2.32382, 3.50944, 3.39548, 3.43384, 2.76083	A:TYR408 (Pi‐Sigma) A:THR412 (Pi‐Sigma) A:TRP386 (Pi–Pi T‐shaped) A:PHE390 (Pi–Pi T‐shaped) A:CYS118 (Alkyl) A:CYS118 (Pi‐Alkyl) A:ALA122 (Pi‐Alkyl) A:VAL115 (Pi‐Alkyl) A:TRP386 (Pi‐Alkyl) A:PHE389 (Pi‐Alkyl) A:PHE390 (Pi‐Alkyl)
	Psoralen	1	A:SER193	2.29328	A:PHE389 (Pi–Pi Stacked) A:PHE198 (Pi–Pi T‐shaped) A:TRP386 (Pi–Pi T‐shaped) A:PHE390 (Pi–Pi T‐shaped) A:CYS118 (Pi‐Alkyl) A:ALA122 (Pi‐Alkyl) A:VAL115 (Pi‐Alkyl)
D_3_	Eticlopride	1	A:TYR373	3.47843	A:ASP110 (Pi‐Anion) A:ILE183 (Alkyl) A:LEU89 (Alkyl) A:VAL86 (Alkyl) A:PHE345 (Pi‐Alkyl) A:TYR373 (Pi‐Alkyl)
	Domperidone	2	A:SER192 A:VAL111	2.45322, 3.64358	A:LEU89 (Pi‐Sigma) A:PHE346 (Pi–Pi T‐shaped) A:HIS349 (Pi–Pi T‐shaped) A:VAL111 (Alkyl) A:ILE183 (Alkyl) A:LEU89 (Alkyl) A:ILE183 (Pi‐Alkyl) A:VAL189 (Pi‐Alkyl) A:VAL111 (Pi‐Alkyl) A:VAL86 (Pi‐Alkyl) A:PHE345 (Pi‐Alkyl)
	Psoralen	1	A:SER196	2.96286	A:ILE183 (Pi‐Sigma) A:PHE345 (Pi–Pi T‐shaped) A:HIS349 (Pi–Pi T‐shaped) A:VAL107 (Pi‐Alkyl) A:VAL111 (Pi‐Alkyl) A:ILE183 (Pi‐Alkyl)
M_1_	Benzeneacetic acid	1	A:GLU397	3.74641	A:TYR404 (Pi–Pi Stacked) A:TYR85 (Pi‐Alkyl)
	Hyosine butyl bromide	1	A:TYR381	3.05741	A:GLU401 (Attractive Charge) A:TYR85 (Pi‐Alkyl) A:TRP101 (Pi‐Alkyl)
	Psoralen	—	—	—	A:TYR404 (Pi–Pi Stacked) A:CYS407 (Pi‐Alkyl)
M_2_	N‐methyl scopolamine	2	A:TYR104 A:PHE181	2.42472, 3.49057	A:TYR426 (Pi–Pi Stacked)
	Hyosine butyl bromide	1	A:ASN419	3.27672	A:TRP422 (Pi‐Sigma) A:TRP422 (Pi‐Alkyl)
	Psoralen	1	A:SER107	2.03749	A:ASP103 (Pi‐Anion) A:TRP400 (Pi–Pi T‐shaped) A:TYR426 (Pi–Pi T‐shaped) A:CYS429 (Pi‐Alkyl)
M_3_	Tiotropium	4	A:TYR506 A:LYS522 A:CYS220 A:CYS220	2.97171, 2.65989, 3.55649, 3.49067	A:TYR529 (Pi‐Cation) A:ILE222 (Pi‐Alkyl) A:LEU225 (Pi‐Alkyl) A:PRO228 (Pi‐Alkyl)
	Hyosine butyl bromide	1	A:LYS522	2.28684	A:TRP525 (Pi‐Cation) A:TYR529 (Pi–Pi Stacked) A:LEU225 (Alkyl)
	Psoralen	—	—	—	A:TYR529 (Pi–Pi Stacked) A:ILE222 (Alkyl)
M_4_	Tiotropium	3	A:TYR89 A:TYR89 A:THR196	3.75603, 3.75639, 2.94447	A:TYR439 (Pi‐Cation) A:ILE187 (Pi‐Alkyl) A:LEU190 (Pi‐Alkyl)
	Hyosine butyl bromide	1	A:TYR416	2.49105	A:TRP435 (Pi–Pi T‐shaped) A:TYR89 (Pi‐Alkyl) A:TRP108 (Pi‐Alkyl) A:TYR439 (Pi‐Alkyl)
	Psoralen	2	A:ASP112 A:SER116	3.51974, 2.54585	A:CYS442 (Pi‐Sulfur) A:TYR113 (Pi–Pi Stacked) A:TRP413 (Pi–Pi Stacked) A:TYR439 (Pi–Pi Stacked) A:TRP413 (Pi–Pi Stacked)
M_5_	Tiotropium	—	—	—	A:TYR481 (Pi‐Sigma) A:TRP477 (Pi–Pi Stacked) A:LEU188 (Amide‐Pi Stacked) A:LEU188 (Pi‐Alkyl)
	Hyosine butyl bromide	2	A:TYR111 A:TYR458	2.46166, 2.93501	A:TRP477 (Pi–Pi T‐shaped) A:VAL474 (Alkyl) A:TRP477 (Pi‐Alkyl)
	Psoralen	—	—	—	A:TRP455 (Pi–Pi T‐shaped) A:TYR458 (Pi–Pi T‐shaped) A:TYR481 (Pi–Pi T‐shaped) A:CYS484 (Pi‐Alkyl)

Abbreviation: HB, hydrogen bond.

For the 5‐HT_3A_ receptor, the co‐crystal ligand (GRN) and OND did not form HBs, relying primarily on hydrophobic interactions such as Pi–cation and Pi–alkyl interactions. In contrast, PSN formed two HBs with residues TYR64 and LYS127, with bond lengths of 2.95 and 2.88 Å, respectively. Additionally, PSN engaged in multiple Pi–type interactions (Pi‐cation, Pi‐sigma, Pi–Pi T‐shaped), indicating a more stabilized binding conformation compared to the reference ligands.

In the D_2_ receptor, DOM exhibited strong binding supported by five HBs, whereas the co‐crystal ligand RPD formed only one HB. PSN also formed a single HB with SER193 (2.29 Å), along with several Pi–Pi stacked and Pi–alkyl interactions involving key residues such as PHE389, TRP386, and PHE390. Importantly, PSN shared several common binding residues with DOM, suggesting a similar binding orientation despite fewer HBs. For the D_3_ receptor, PSN formed one HB (SER196, 2.96 Å), comparable to the co‐crystal ligand ETI (one HB), but fewer than DOM (two HBs). However, PSN maintained stable interactions through hydrophobic contacts, including Pi–Pi and Pi–alkyl interactions with residues such as PHE345 and VAL111.

In the M_1_ receptor, PSN did not form HBs, unlike both the co‐crystal ligand and HYS, each of which formed one HB. Nevertheless, PSN maintained stability via Pi–Pi stacked and Pi–alkyl interactions (TYR404 and CYS407), indicating that nonhydrogen bonding interactions contribute significantly to its binding. For the M2 receptor, PSN formed one HB with SER107 (2.03 Å), which is shorter and potentially stronger than that observed with HYS. Additionally, PSN exhibited diverse interactions, including Pi–anion and Pi–Pi interactions, enhancing binding stability. In the M3 receptor, PSN did not form HBs, unlike TIO (four HBs) and HYS (one HB), which explains its comparatively lower binding affinity. However, PSN still interacted via hydrophobic contacts such as Pi–Pi stacking. For the M4 receptor, PSN formed two HBs (ASP112 and SER116), whereas HYS formed only one and TIO formed three. PSN also exhibited extensive Pi–Pi stacked and Pi–sulfur interactions, suggesting strong stabilization within the binding pocket. Finally, for the M5 receptor, PSN did not form HBs, similar to TIO, but engaged in multiple Pi–Pi T‐shaped and Pi–alkyl interactions with residues such as TYR458 and TRP455. In contrast, HYS formed two HBs, indicating a difference in binding mechanism.

Overall, PSN demonstrated a diverse interaction profile, combining hydrogen bonding (in selected receptors) with extensive hydrophobic and Pi‐type interactions. Although it generally formed fewer HBs compared to standard drugs like DOM or TIO, PSN compensated through strong hydrophobic interactions and shared binding residues, supporting its stable binding within receptor active sites. These findings further reinforce the multi‐target interaction capability of PSN, which may underlie its observed pharmacological effects. Figure 3 represents the 3D and corresponding 2D interaction diagrams showing the binding orientation of PSN and standard drugs (DOM, OND, HYS) within the active sites of 5‐HT_3_, D_2_, and muscarinic receptors.

FIGURE 3Molecular docking interaction analysis of selected ligands with emesis‐related receptors. Representative 3D and corresponding 2D interaction diagrams showing the binding orientation of psoralen (PSN) and standard drugs (DOM, OND, HYS) within the active sites of 5‐HT_3_, D_2_, and muscarinic receptors. Hydrogen bonds, hydrophobic interactions, and key amino acid residues involved in ligand stabilization are highlighted. These interactions support the predicted binding affinities and provide structural insight into the possible receptor‐mediated antiemetic mechanisms.

**Figure:**
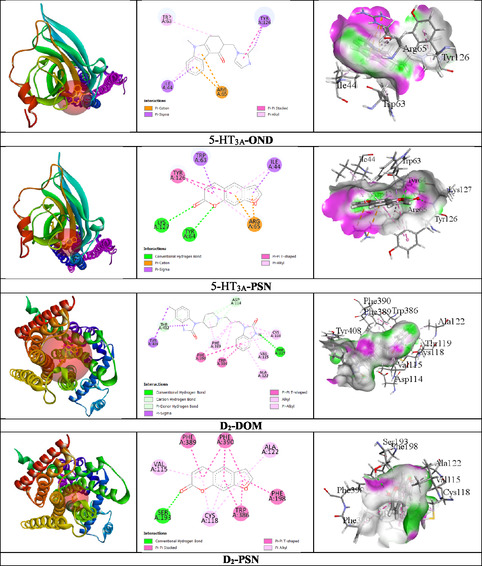

**Figure:**
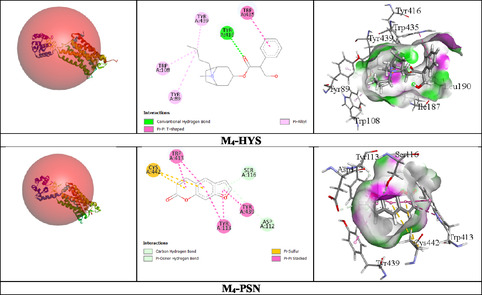

#### 3.2.3 Prediction of Drug‐Likeness and Pharmacokinetics

**FIGURE 4:**
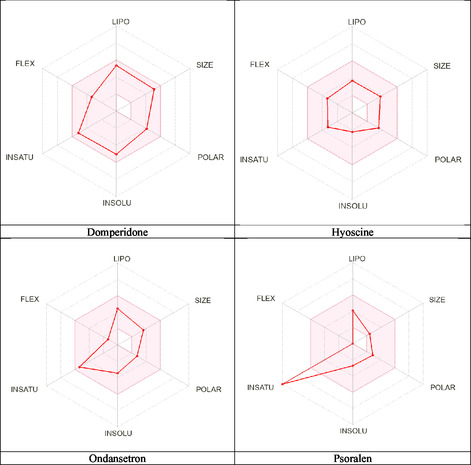
SwissADME bioavailability radar plots of psoralen and reference antiemetic drugs. The radar charts represent six key physicochemical parameters influencing oral bioavailability: lipophilicity (LIPO), molecular size (SIZE), polarity (POLAR), solubility (INSOLU), flexibility (FLEX), and saturation (INSATU). The pink shaded region indicates the optimal physicochemical space for oral drug‐likeness. Compounds falling within this region are predicted to possess favorable pharmacokinetic characteristics.

A drug candidate's ability to resemble a medication is crucial, and this involves turning a chemical substance into a pharmaceutical and evaluating its pharmacokinetics. In silico ADMET has a significant role in drug development and discovery. Along with being effective against the therapeutic target, a high‐quality drug candidate should also have appropriate ADMET properties at therapeutic levels. Molecular weight (MW), hydrogen bond donors (HBD), hydrogen bond acceptors (HBA), molar refractivity (MR), and log *P* are the primary metrics used to assess drug‐likeness. According to our research, every drug has a molecular weight under 500 Daltons. HBD (≤5) and HBA (≤10) are both within the limit based on Lipinski's rule of five. Additionally, the data demonstrated that whereas DOM is similarly soluble in water, HYS, OND, and PSN are not. In the gastrointestinal system, all medications are well absorbed. The findings also demonstrated that PSN has every pharmacokinetic and physicochemical characteristic of a molecule that resembles a medication. Although the molecule deviates from the Muegge criteria due to its molecular weight being less than 200, it also satisfies the Lipinski, Ghose, Egan, and Veber requirements to guarantee drug‐likeness. Other metrics, such as P‐gp substrate, TPSA, CYP2C19 inhibitor, BBB permeability, and bioavailability score of PSN and reference drugs, are included in (Table 6) and illustrated graphically in Figure 4.

**TABLE 6:** Pharmacokinetic and drug‐likeness properties of Psoralen predicted by SwissADME.

Parameter	Domperidone	Hyosine butyl bromide	Ondansetron	Psoralen
**Physicochemical properties**				
MF	C_22_H_24_ClN_5_O_2_	C_17_H_21_NO_4_	C_18_H_19_N_3_O	C_11_H_6_O_3_
MW	425.91	303.35	293.36	186.16
No. of heavy atoms	30	22	22	14
No. of aromatic heavy atoms	18	6	14	13
No. of H‐bond acceptors	3	5	2	3
No. of H‐bond donors	2	1	0	0
MR	124.08	83.48	87.39	52.26
TPSA, Å^2^	78.82	62.3	39.82	43.35
**Solubility**				
Solubility (water)	Moderately soluble	Soluble	Soluble	Soluble
**Lipophilicity**				
Log Po/w (XLOGP3)	3.90	0.98	2.29	1.67
**Pharmacokinetics**				
GI absorption	High	High	High	High
BBB permeant	Yes	No	Yes	Yes
P‐gp substrate	Yes	No	Yes	No
CYP2C19 inhibitor	Yes	No	Yes	No
Log Kp (skin permeation) (cm/s)	−6.13	−7.45	−6.46	−6.25
**Drug likeness**				
Lipinski	Yes; 0 violation	Yes; 0 violation	Yes; 0 violation	Yes; 0 violation
Ghose	Yes	Yes	Yes	Yes
Veber	Yes	Yes	Yes	Yes
Egan	Yes	Yes	Yes	Yes
Muegge	Yes	Yes	Yes	No; 1 violation: MW < 200
BIO score	0.55	0.55	0.55	0.55

#### 3.2.4 Toxicity Prediction

The toxicological investigation of small‐molecule compounds is critical for determining their safety and usefulness in both preclinical animal models and prospective human applications. The online tool ProTox‐3.0 was used to predict the toxicity profiles of the chosen medication candidates. In silico study classified DOM, HYS, and PSN as toxicity class 4 (possibly harmful if consumed; LD_50_ between 300 and 2000 mg/kg), while OND was assigned to toxicity class 3 (toxic if ingested; LD_50_ between 50 and 300 mg/kg). Regarding carcinogenic potential, all reference drugs were projected to be inactive; however, PSN displayed an active reaction, indicating potential carcinogenicity. Endpoint projections were used to further analyze the toxicity risk. Both HYS and PSN demonstrated no hepatotoxicity, immunotoxicity, mutagenicity, or cytotoxicity. In contrast, DOM was anticipated to be immunotoxic, whereas OND had mutagenic potential. Other investigated toxicity objectives for these drugs were declared ineffective. Table 7 lists the various toxicity parameters, as well as their status or levels, for our chemical compounds.

**TABLE 7:** Toxicity profiling of the test sample and reference antiemetic drugs.

Properties	Parameter	Domperidone	Hyosine butyl bromide	Ondansetron	Psoralen
Toxicity	LD50 (mg/kg)	715	1275	95	322
	Toxicity Class	4	4	3	4
	Hepatotoxicity	Inactive	Inactive	Inactive	Inactive
	Carcinogenicity	Inactive	Inactive	Inactive	Active
	Immunotoxicity	Active	Inactive	Inactive	Inactive
	Mutagenicity	Inactive	Inactive	Active	Inactive
	Cytotoxicity	Inactive	Inactive	Inactive	Inactive

## 4 Discussion

Vomiting, also known as emesis, is a finely coordinated reaction that helps the body rid itself of dangerous chemicals [5]. The brainstem central pattern generator (CPG), NTS, and area postrema are the main brainstem regions that mediate this response [19]. Both central and peripheral routes provide input to these centers [20]. The vagal and splanchnic afferents that project to the emetic centers are activated by peripheral stimuli, such as poisons or irritants in the gastrointestinal system [21]. Since the postrema lacks a blood‐brain barrier, circulating emetogens that cause central stimuli directly excite the region [5]. In triggering this reaction, neurotransmitters such as substance P (NK1 receptors), histamine (H_1_ receptors), acetylcholine (M1 receptors), serotonin (5‐HT_3A_ receptors), and dopamine (via D_2_ receptors) are essential [22, 23]. Copper sulfate pentahydrate (CuSO_4_ · 5H_2_O), a well‐known peripherally acting emetogen that is frequently employed in experimental models to imitate gastrointestinal‐induced emesis, was utilized in this investigation to produce emesis in chicks [13, 24]. By directly irritating the gastrointestinal mucosa, CuSO_4_ · 5H_2_O is known to cause vomiting [25]. This triggers the activation of vagal afferent neurons, which transmit signals to the region postrema and NTS, which are important parts of the central emetic circuitry found in the medulla oblongata [26, 27]. Through the CPG, a network that starts the motor act of vomiting or retching, these areas coordinate emetic reactions [28]. Copper salts mostly have a peripheral emetogenic effect, activating enterochromaffin cells and releasing serotonin(5‐HT), which in turn activates 5‐HT_3A_ receptors on vagal terminals [21, 29]. CuSO_4_ · 5H_2_O is especially well‐suited for assessing drugs that target peripheral emetic pathways because of this mechanism [30].

The chick model, which is often used for antiemetic screening, has a number of benefits [31]. Chicks respond to both central and peripheral stimuli with measurable and obvious emetic activity, in contrast to rats, which are physically incapable of vomiting [5]. The model is pharmacologically relevant and predictive for human emetic reactions since it is responsive to a variety of common antiemetic drugs, such as dopamine antagonists, serotonin receptor antagonists, and anticholinergics, and sensitive to a wide range of emetogens [6, 32]. Its translational value is further enhanced by the fact that the neurochemical processes driving emesis in chicks, namely the involvement of D_2_, 5‐HT_3A_, and muscarinic receptors, are similar to those observed in mammals [33]. The current findings with PSN show that, in light of these characteristics, the CuSO_4_ · 5H_2_O‐induced chick emesis model offers a reliable and validated platform for the in vivo screening of new antiemetic drugs.

**FIGURE 5:**
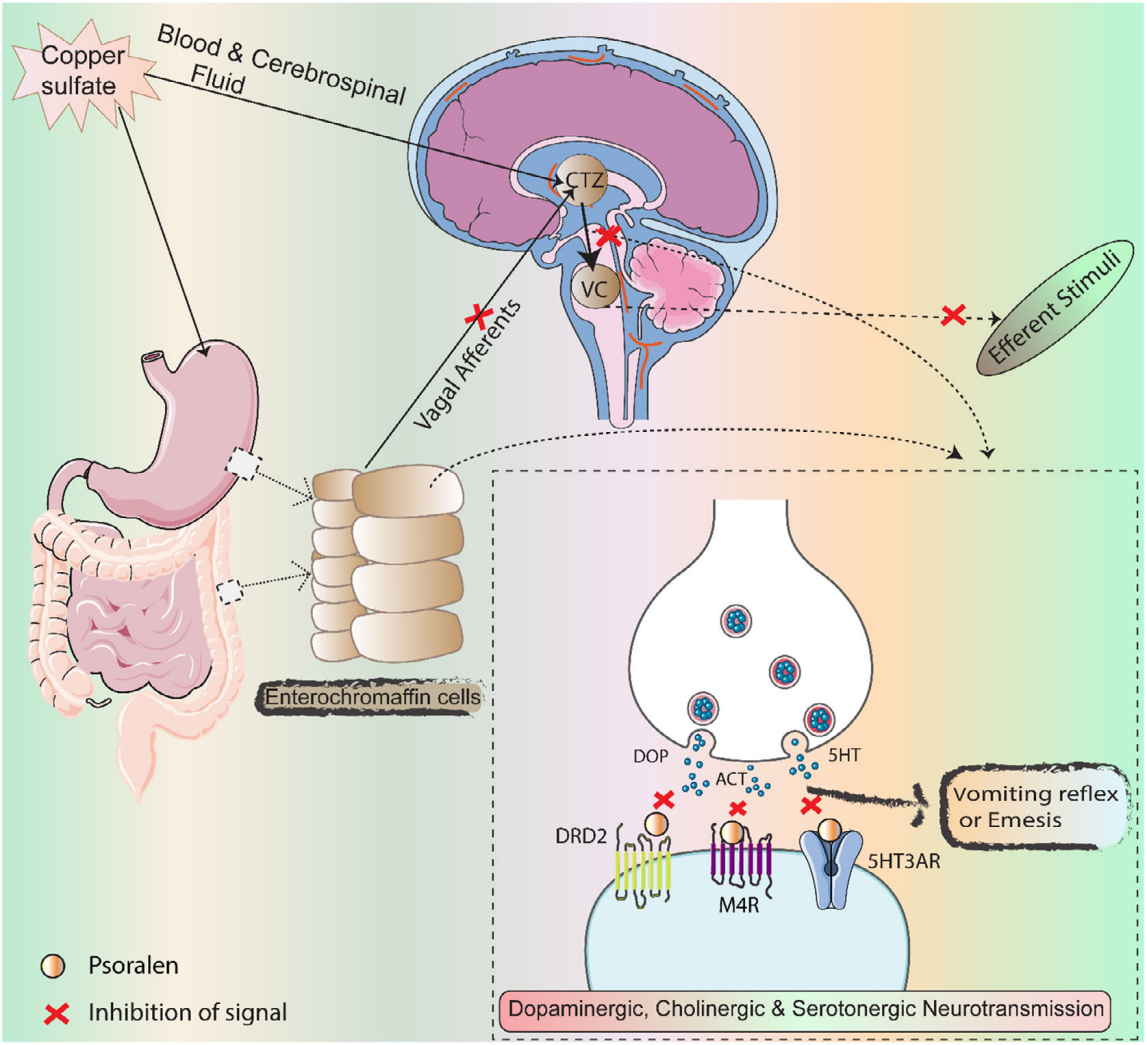
Proposed multi‐target antiemetic mechanism of psoralen (PSN) in comparison with standard antiemetic drugs. The schematic diagram illustrates the possible involvement of dopaminergic (D_2_), serotonergic (5‐HT_3A_), and cholinergic (M_1_–M_5_) pathways in copper sulfate–induced emesis. Standard drugs selectively block individual receptors (DOM: D_2_ antagonist; OND: 5‐HT_3A_ antagonist; HYS: muscarinic antagonist), whereas PSN may exert modulatory effects through multi‐receptor interactions, potentially contributing to its dose‐dependent reduction in retching frequency and increased latency observed in vivo studies.

The test chemical PSN showed a dose‐dependent antiemetic effect in the chick model, according to the in vivo results. The effects of common antiemetic medications, including DOM, OND, and HYS, were outperformed by the highest dose, PSN‐20, which demonstrated the biggest decrease in the frequency of retches (10.8 ± 1.23) and a considerable rise in the latency time (64.4 ± 2.65 s). The equivalent retching frequencies for standard medicines were 13.6 ± 1.71, 14.8 ± 1.98, and 23 ± 1.92 s, whereas the corresponding latency values were 45.8 ± 2.59, 57.2 ± 3.76, and 55.8 ± 5.14 s. It's interesting to note that combination treatment with PSN‐20 and OND‐5 had the most benefit, reducing retches to 10.8 ± 1.27 and latency to 79.8 ± 4.21 s, suggesting a possible synergistic interaction. These findings are in line with previous research emphasizing the advantages of addressing several emetogenic pathways. While DOM, a D_2_ receptor blocker, and HYS, an antimuscarinic drug, are used to treat motion sickness and other causes of nausea, OND, a 5‐HT_3A_ antagonist, is useful in treating nausea brought on by chemotherapy. The higher effectiveness of PSN, especially at 20 mg/kg, raises the possibility that it has unique modes of action or interacts with many neurotransmitter systems. PSN‐20 and PSN‐20 + OND‐5 groups demonstrated the greatest reduction in retching, both exhibiting a 65.60% drop, surpassing the results of separate conventional treatments. The possible mechanism of PSN is presented in Figure 5.

Molecular docking analyzes drug–receptor interactions and predicts how small‐molecule drug candidates bind to their protein targets. This process enables the prediction of binding affinity and the identification of critical interactions, including hydrogen bonds and hydrophobic contacts [34, 35]. Computational studies enhance the screening and design of drug candidates with increased precision and efficacy, thereby lowering the expenses related to new drug development [36]. The molecular docking and interaction analyses suggest that PSN may exert antiemetic effects through multi‐receptor modulation involving dopaminergic, serotonergic, and muscarinic pathways. Among the investigated targets, PSN demonstrated the strongest interaction with the D_2_ receptor, where it formed a hydrogen bond with SER193 together with multiple Pi‐mediated hydrophobic interactions involving TRP386, PHE389, PHE390, CYS118, and VAL115. Several of these interacting residues were also shared with DOM, indicating partial similarity in receptor‐binding orientation and suggesting that dopaminergic pathway modulation may contribute to the observed antiemetic activity of PSN.

PSN also exhibited stable interactions with muscarinic receptor subtypes, particularly M_4_ and M_5_ receptors, primarily through aromatic hydrophobic contacts rather than extensive hydrogen bonding. In the M_5_ receptor, PSN interacted through multiple Pi–Pi T‐shaped interactions involving TRP455, TYR458, and TYR481, indicating stable accommodation within the receptor binding pocket. Similarly, the interaction profile observed in the 5‐HT_3_ receptor, characterized by hydrogen bonding and Pi‐type interactions, supports the possible involvement of serotonergic signaling modulation. Collectively, these findings indicate that PSN may stabilize receptor complexes through coordinated hydrophobic and aromatic interactions, thereby supporting a potential multi‐target mechanism underlying its antiemetic effects. However, as docking analysis provides only predictive structural insight, further functional and mechanistic studies are required to validate receptor‐specific pharmacological activity.

In recent years, modern antiemetic therapy has expanded to include neurokinin‐1 (NK1) receptor antagonists such as aprepitant, which inhibit substance P–mediated signaling within the central emetic pathway. NK1 antagonists are particularly effective in chemotherapy‐induced nausea and vomiting. In contrast, the copper sulfate–induced emesis model primarily involves peripheral serotonergic activation and dopaminergic modulation. Therefore, the antiemetic activity observed for PSN may be more closely associated with serotonergic and dopaminergic receptor interactions rather than NK1 blockade, indicating a pharmacological profile distinct from NK1‐targeted agents.

The in vivo findings demonstrated that PSN significantly reduced retching frequency (65.60% inhibition at 20 mg/kg), indicating modulation of emesis‐related neurophysiological pathways. Computational docking revealed that PSN exhibited its strongest predicted binding affinity toward the D_2_ receptor (−8.8 kcal/mol), a key mediator of dopaminergic emetic signaling, with moderate affinities toward 5‐HT_3A_ (−7.7 kcal/mol) and muscarinic receptors, including M_5_ (−8.1 kcal/mol).

Emesis is regulated through integrated signaling between peripheral vagal afferents and central emetic nuclei, particularly the area postrema, CTZ, and NTS, involving dopaminergic, serotonergic, and cholinergic pathways. Although molecular docking does not establish functional antagonism, the concordance between the predicted receptor‐binding profile and the observed in vivo antiemetic efficacy suggests that PSN may exert its pharmacological effect through coordinated modulation of multiple emesis‐related signaling pathways, with D_2_ receptor interaction potentially playing a contributory role. This integrated mechanism may underlie the dose‐dependent reduction in retching observed in the chick model.

Drug‐likeness denotes the physicochemical resemblance of compounds to established drugs. This concept serves to eliminate undesirable properties, with the ultimate goal of optimizing the drug development process [37]. It was initially determined through the assessment of physicochemical properties to identify candidates suitable for oral administration [38]. Parameters such as the Lipinski rule [39], Ghose rule [40], Veber rule [41], Egan rule [42], and Muegge rule [43] are applied to assess the pharmacokinetic features that contribute to the drug‐likeness of any molecule that has been virtually screened. In our study, all the drugs adhered to these rules; however, PSN violated the Muegge rule because its molecular weight is less than 200. All compounds demonstrated high gastrointestinal (GI) absorption and exhibited a comparable bioavailability score of 0.55. This shows that the tested chemical may attain therapeutic plasma concentrations similar to the standard, confirming its potential as a successful oral contender.

The drug development procedure is a great difficulty in the drug manufacturing industry as it requires a large amount of time and money to proceed through all the phases of creating a new medicine [44]. This time‐consuming procedure can be shortened by using the in silico approach for toxicological prediction, which reduces the need for animals and saves cost [45]. In silico toxicology is used to forecast particular hazards, such as mutagenicity or organ toxicities, based on a computational approach, which may be developed by structural warnings or machine learning methods, depending on the stage of drug development [45]. In our present study, PSN demonstrated no toxic effects in terms of hepatotoxicity, immunotoxicity, mutagenicity, and cytotoxicity. However, it did exhibit toxicity effects in terms of carcinogenicity. In contrast, the standard drug DOM exhibits immunotoxicity, HYS showed no toxicity, and OND exhibits mutagenicity.

PSN is a well‐established photosensitizing compound clinically utilized in PSN plus ultraviolet A (PUVA) therapy for dermatological disorders [46, 47, 48, 49]. Its phototoxic effect is mediated through UVA‐induced DNA interstrand crosslink formation, which underlies both its therapeutic efficacy and its associated mutagenic and carcinogenic risks during prolonged UV exposure [50, 51]. In the present study, animals were not exposed to ultraviolet radiation, and PSN was administered acutely (5–20 mg/kg), conditions that differ substantially from chronic PUVA therapy associated with cumulative oncogenic risk. The carcinogenicity signal predicted by in silico toxicity analysis therefore warrants cautious interpretation. Computational models primarily identify structural alerts linked to potential carcinogenicity rather than experimentally confirmed tumorigenic outcomes. Accordingly, the observed prediction represents a theoretical concern under long‐term or UV‐activated conditions rather than evidence of carcinogenicity at the doses used in this study.

In addition to phototoxic considerations, PSN has been reported to interact with cytochrome P450 enzymes, particularly CYP3A4, potentially altering drug metabolism and increasing the risk of drug–drug interactions when co‐administered with other medications. Notably, no observable acute toxicity was detected at the highest tested dose (20 mg/kg) in the present in vivo study. Although the median lethal dose (LD_50_) was not determined here, previously reported LD_50_ values in animal models indicate a substantially higher toxic threshold than the effective antiemetic doses applied, suggesting the presence of a preliminary therapeutic window. Nevertheless, from a translational perspective, comprehensive chronic toxicity, genotoxicity, pharmacokinetic, and dose‐escalation studies in mammalian models remain essential to define dose‐dependent risk, establish an accurate therapeutic index, and ensure long‐term safety prior to clinical application.

All things considered, the study shows that PSN may strong antiemetic candidate. Its effectiveness not only equaled but also surpassed existing conventional therapy in certain areas. Strong evidence supports the compound's potential clinical use, particularly in situations where combination therapy is required to target multiple emetic pathways, as it can significantly reduce the number of retching episodes and delay the onset of emesis in a dose‐dependent manner. Despite promising in vivo and in silico findings, several limitations should be acknowledged. The precise mechanistic basis of PSN's antiemetic action remains unclear and requires further neuropharmacological investigation. The study employed a single animal model, and translational relevance to human physiology warrants validation in mammalian systems. Receptor interaction analysis was based solely on molecular docking, which provides static, predictive binding estimates without accounting for receptor dynamics or binding stability, necessitating further validation through molecular dynamics and free energy analyses. Additionally, the pharmacokinetic profile, long‐term safety, and metabolic behavior of PSN were not evaluated.

Therefore, investigations of receptor‐binding and neurotransmitter interactions should be the main focus of future study in order to clarify the molecular processes of PSN. Pharmacokinetic and toxicological analyses will also be required to determine whether it is suitable for clinical development. The compound's broad‐spectrum antiemetic potential might be further confirmed by extending in vivo testing to include mammalian models and other emetogenic triggers, such as motion or chemotherapeutic drugs. PSN could be a useful supplement to the current antiemetic treatment arsenal if these investigations are effective.

## 5 Conclusion

The findings of this study reveal that PSN effectively lowers the frequency of retching and greatly increases delay in CuSO_4_ · 5H_2_O‐induced emesis in chicks. In silico investigations further support the promise of PSN, indicating positive drug‐likeness, excellent pharmacokinetic features, and especially substantial binding affinities, particularly toward the D_2_ dopamine receptor and the M_4_‐M_5_ subtype of muscarinic receptors. Moreover, when provided in conjunction with proven antiemetic medicines, PSN increases anti‐retching effectiveness. Coadministration with the 5‐HT_3A_ receptor antagonist OND, in particular, resulted in a substantial decrease in CuSO_4_ · 5H_2_O‐induced retching. In addition, the predicted carcinogenic signal from in silico toxicity analysis and the established photosensitizing nature of PSN warrant careful safety consideration. Although phototoxic activation was not relevant under the present experimental conditions, these intrinsic properties highlight the importance of comprehensive toxicological, phototoxicity, and long‐term safety evaluations before any potential clinical translation of PSN as an antiemetic agent, further research is needed to clarify the interactions between neurotransmitters and pharmacokinetics, as well as the toxicology of PSN.

## Author Contributions

**Kishor Chandra** and **Khadija Akter:** conceptualization. **Sharif Uddin Bahar:** methodology. **Kishor Chandra, Mst. Muslima Khatun, Salehin Sheikh, Progna Saha Puja**, and **Fazley Rohan**: experimental observation. **Sharif Uddin Bahar** software. **Mst. Muslima Khatun, Md Shimul Bhuia**, and **Mohammed Alfaifi:** validation. **Khadija Akter, Md Shimul Bhuia, Faisal H. Altemani, Faisal Alsenani**, and **Muhammad Torequl Islam:** formal analysis. **Md Shimul Bhuia, Faisal Alsenani,** **Kishor Chandra:** data curation. **Mohammed Alfaifi:** funding acquisition. **Kishor Chandra, Sharif Uddin Bahar, Khadija Akter,** **Mst. Muslima Khatun:** writing – original draft preparation. **Kishor Chandra, Sharif Uddin Bahar, Salehin Sheikh, Mst. Muslima Khatun**, and **Samiran Sadhukhan:** writing – review and editing. **Salehin Sheikh:** mechanisms drawing. **Khadija Akter, Salehin Sheikh**, and **Muhammad Torequl Islam:** supervision. **Khadija Akter, Salehin Sheikh**, and **Muhammad Torequl Islam:** project administration.

## Funding

This work was supported by Deanship of Research and Graduate Studies at King Khalid University for funding this work through the large group Research Project under grant number (RGP1‐112‐46).

## Ethics Statement

This study was approved by the GSTU Animal Ethics Committee (#GSTU‐pharmacy‐19PHR022). Furthermore, all processes were carried out in compliance with the relevant laws and regulations, and they were all reported using the ARRIVE principles (https://arriveguidelines.org).

## Consent to Participate

We hereby confirm that this study did not involve any human participants. All experiments and procedures were conducted on animals in accordance with ethical guidelines and institutional regulations.

## Consent to Publish

All authors have read and agreed to the published version of the manuscript.

## Conflicts of Interest

The authors declare no conflicts of interest.

## Data Availability

Data will be available from the authors on request.
